# Influence of Generated Defects by Ar Implantation on the Thermoelectric Properties of ScN

**DOI:** 10.1021/acsaem.2c01672

**Published:** 2022-08-23

**Authors:** Razvan Burcea, Jean-François Barbot, Pierre-Olivier Renault, Dominique Eyidi, Thierry Girardeau, Marc Marteau, Fabien Giovannelli, Ahmad Zenji, Jean-Michel Rampnoux, Stefan Dilhaire, Per Eklund, Arnaud le Febvrier

**Affiliations:** † Institute PPRIME, CNRS, Université de Poitiers-ENSMA, UPR 3346, SP2MI, TSA 41123, 86073 Poitiers cedex 9, France; ‡ Laboratoire GREMAN, CNRS, Université de Tours, UMR 7347, 41029 Blois cedex, France; § Laboratoire LOMA, CNRS, Université de Bordeaux, UMR 5798, 33405 Talence, France; ∥ Department of Physics, Chemistry and Biology (IFM), Linköping University, SE-581 83 Linköping, Sweden

**Keywords:** nitride, thermoelectric, ion implantation, defects, phonon scattering

## Abstract

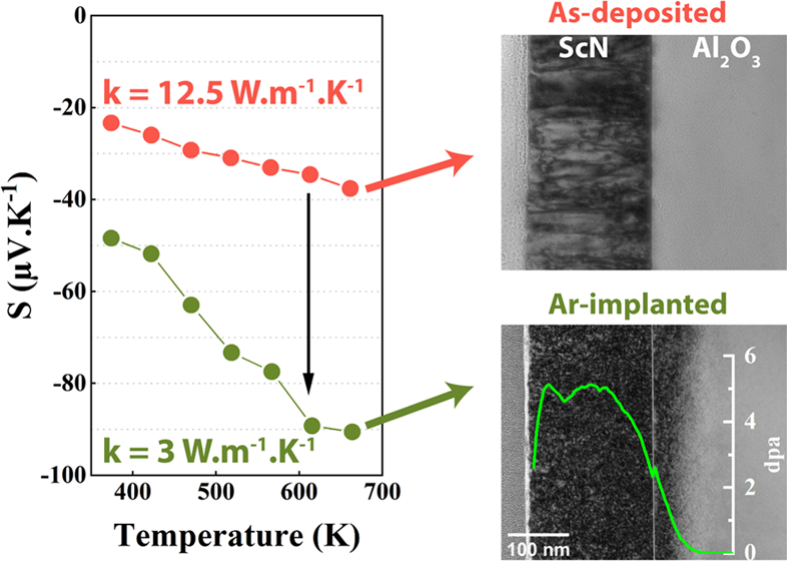

Nowadays, making thermoelectric materials more efficient in energy conversion is still a challenge. In this work, to reduce the thermal conductivity and thus improve the overall thermoelectric performances, point and extended defects were generated in epitaxial 111-ScN thin films by implantation using argon ions. The films were investigated by structural, optical, electrical, and thermoelectric characterization methods. The results demonstrated that argon implantation leads to the formation of stable defects (up to 750 K operating temperature). These were identified as interstitial-type defect clusters and argon vacancy complexes. The insertion of these specific defects induces acceptor-type deep levels in the band gap, yielding a reduction in the free-carrier mobility. With a reduced electrical conductivity, the irradiated sample exhibited a higher Seebeck coefficient while maintaining the power factor of the film. The thermal conductivity is strongly reduced from 12 to 3 W·m^–1^·K^–1^ at 300 K, showing the influence of defects in increasing phonon scattering. Subsequent high-temperature annealing at 1573 K leads to the progressive evolution of these defects: the initial clusters of interstitials evolved to the benefit of smaller clusters and the formation of bubbles. Thus, the number of free carriers, the resistivity, and the Seebeck coefficient are almost restored but the mobility of the carriers remains low and a 30% drop in thermal conductivity is still effective (*k*_total_ ∼ 8.5 W·m^–1^·K^–1^). This study shows that control defect engineering with defects introduced by irradiation using noble gases in a thermoelectric coating can be an attractive method to enhance the figure of merit of thermoelectric materials.

## 1 Introduction

Defects, inevitable in materials, are classified as point defects (vacancy, interstitial, impurity), linear (dislocation), planar (stacking fault, grain boundary), and volumetric defects (from nanocavity up to microcrack). They are known to influence the properties of materials by themselves and by interacting with each other. Some specific defects can also be introduced deliberately, particularly after the material has been subjected to irradiation/implantation or plastic deformation. For use, impurities are introduced into semiconductors to tailor their electrical conductivity σ. On the contrary, in metals, such impurities can reduce the conductivity by acting as obstacles to a smooth carrier flow and play a key role in extrinsic phonon scattering in lowering the thermal conductivity *k*_total_. The ion implantation technique in which specific elements can be selected and then accelerated toward a target material is widely used in several fields such as the microelectronic industry, surface modification, or to simulate the behavior of materials under a harsh environment (nuclear applications). The process results in the generation of a large concentration of Frenkel pairs via the collision cascades, which tend to recombine and/or condense to form various types of defects depending on the experimental conditions such as the species of implanted atoms, the fluence, the incident energy, and the thermal budget. An advantage of ion implantation is that the as-induced changes in physical properties can be manipulated repeatedly, which makes it a suitable technique for dealing with the transport properties of thin-film thermoelectric (TE) materials. Indeed, the efficiency of a TE material to convert heat to electricity is governed by its figure of merit *ZT* = *S*^2^ σ*T*/*k*_total_, where *T* is the absolute operating temperature, *S* is the Seebeck coefficient, σ is the electrical conductivity, and *k*_total_ is the thermal conductivity being the sum of the lattice and electronic thermal conductivity (*k*_lat_ + *k*_e_). The nanoscale defects generated by implantation (may be followed by high-temperature annealing) could be used as a scattering means for short-wavelength phonons, leading to a reduction in *k*_lat_. Besides, defects are currently seen as a promising way forward in a strategy to improve the efficiency of TE materials.^1^ The vacancy engineering strategy has been used to create dislocations in PbTe for reducing the lattice thermal conductivity.^2^ Similarly, the introduction of excess Cu atoms in CuSe thin films reduces the Cu vacancies and introduces additional scattering centers, increasing the *ZT*.^3^ It should be noted that other strategies for enhancing *ZT* have been developed, for example, quantum confinement,^4,5^ the use of antisite defects in ZrNiSn half-Heusler alloys, or incorporation nanoinclusions in the TE matrix.^6,7^ The controlled introduction of nanoscale defects using ion implantation can also be part of the strategies to scatter phonons reducing the thermal conductivity in thin-film TE materials.

This ion beam technique has recently been used in TE films to modify the electronic transport properties in relation to microstructural modifications. For example, the S^+^ implantation in Bi_0.5_Sb_1.5_Te_3_ increases the thermoelectric power factor, PF = *S*^2^σ, via the generation of carriers due to the TeBi antisites.^8^ Similarly, the *N* implantation in SrTiO_3_ creates mainly oxygen vacancies acting as carrier donors while decreasing the grain size, leading to an enhancement in the PF value.^9^ In CoSb_3_, the Fe^+^ implantation changes the conductivity type due to the creation of vacancies, and an increase of PF by more than a factor of 10 is reported.^10^ All of these authors highlight defect engineering in the applications of TE devices by an increase of the PF. It should be noted, however, that it is mainly the direct or indirect doping effect that is at the origin of the modifications of the electronic properties. These are strongly dependent on the implantation conditions, and in particular on the dose. Ion implantation can also lead to the formation of distinct phases, such as the Ag_2_Te phases in Ag-implanted PbTe, resulting in an increase in the Seebeck coefficient.^11^ In summary, implantation in TE films was used as a means of controlling charge carrier properties but not as a means of introducing lattice defects to strength phonon scattering.

Scandium nitride (ScN) is an n-type semiconductor with suitable properties such as a high carrier concentration in the range of 10^18^–10^22^ cm^–3^ and a low electrical resistivity of about 300 μΩ·cm, leading to an appreciable PF of about 3 × 10^–3^ W m^–1^ K^–2^ at 600 K.^12,13^ However, due to its high thermal conductivity, the overall *ZT* is limited, in the range of 0.2–0.3.^13−15^ The total thermal conductivity, mainly dominated by the lattice conductivity *k*_lat_,^16^ is found to be in the range of 10–12 W m^–1^ K^–1^ at room temperature (RT) and decreases with increasing temperature due to Umklapp scattering (7–8 W m^–1^ K^–1^ at 500 K).^15^ Attempts were made to reduce the thermal conductivity by alloy scattering such as the introduction of Nb (∼10 atom %), which led to a large decrease of the thermal conductivity, down to 2.2 W m^–1^ K^–1^ but deteriorated the Seebeck effect.^17^ More recently, it has been shown that defect introduction using Mg-dopant implantation leads to an increase in the Seebeck coefficient coupled with a drop in the thermal conductivity *k*_total_, down to 3.2 W m^–1^ K^–1^ for the ScN sample implanted with 2.2 atom % of Mg.^18^ Another study has shown the potential of Li^+^-implanted ScN for which the thermal conductivity is divided by half in the 300–700 K temperature range.^19^ Defect engineering by ion implantation and other techniques has shown potential for improvement of thermoelectric properties. However, the control of the induced defect in a material is always a challenge in terms of their formation during irradiation/implantation, the type of defect, and their stability with temperature, which is critical for thermoelectric applications.

The underlined idea of the present work is to reduce the lattice thermal conductivity, *k*_lat_, of ScN by introducing a network of lattice defects (acting as phonons scattering centers) via the ion implantation while trying to keep the power factor constant and thereby improve the *ZT* value. To promote the introduction of defects and minimize the chemical doping effects of the implanted species, a heavy noble gas (NG), argon, was implanted in the ScN thin films. The effects of postimplantation annealing up to 1573 K were also investigated to discuss the implantation-induced defect evolution in relation to changes in thermoelectric properties. In many materials, metals, and semiconductors,^20,21^ the interaction of gas atoms with excess vacancies leads to the formation of nanoscale bubbles that can turn into voids if desorption takes place under subsequent high-temperature annealing. Results show that these Ar-implantation-induced defects modify the physical properties of ScN films by reducing the thermal conductivity while maintaining a roughly constant power factor, thus showing their potential for improving *ZT*.

## 2 Material and Methods

**Figure 1:**
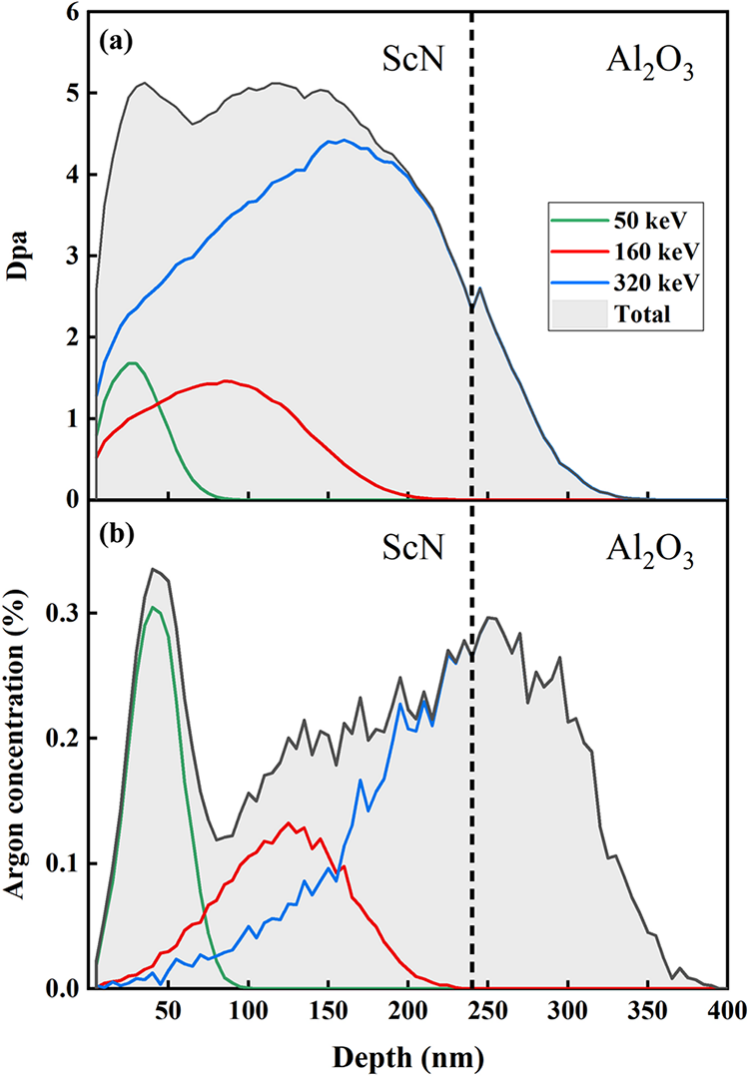
(a) Displacement per atom (dpa) and (b) Ar concentration profiles as the function of depth calculated using the SRIM code. The ScN film was implanted with 320–160–50 keV Ar ions at fluences of 3.5 × 10^15^, 1 × 10^15^, and 1 × 10^15^ cm^–2^, respectively, to obtain a flat damage profile of around 5 dpa throughout the film (gray).

<111> degenerate n-type ScN thin films (thickness ∼ 240 nm) were deposited using dc reactive magnetron sputtering onto Al_2_O_3_ (c-cut) substrates maintained at a temperature of 800 °C (for more details on the growth conditions, see ref (18)). The thin films were then implanted with argon ions, Ar^2+^, at room temperature using the implanter EATON VN3206 at Pprime Institute (Poitiers). The depth profiles of the displaced atoms and implanted ions in the ScN films (density of 4.29 g cm^–3^) were calculated using SRIM 2013 software under the full-damage cascade mode.^22^ To introduce a constant quantity of damage (called displacements per atom: dpa) along the thickness of the film, a multi-implantation protocol was designed. Three implantations with decreasing incident energies of 320 keV (projected ion mean range of *R*_p_ ∼ 200 nm with straggling Δ*R*_p_ ∼ 60 nm), 160 keV (*R*_p_ ∼ 100 nm and Δ*R*_p_ ∼ 35 nm), and 50 keV (*R*_p_ ∼ 35 nm and Δ*R*_p_ ∼ 15 nm) were carried out with fluences of 3.5 × 10^15^, 1 × 10^15^, and 1 × 10^15^ cm^–2^, respectively. These relevant fluences were chosen to implant the ScN film in the called high damage regime, i.e., 5–6 dpa, for which a previous study showed that the impact of defects on thermal conductivity is the most significant.^18^Figure 1 shows the resulting damage distribution (dpa) and argon concentration. As seen, the dpa profile is rather flat in the entire ScN film, around 5 dpa, while the argon concentration is bumpy in the range of 0.1–0.3 atom % and extends deep into the substrate. During implantation, the current beam density was kept below 5 μA·cm^–2^ to avoid any temperature increase. SRIM calculations, which do not consider any dynamic recombination, result in a vacancies/ion ratio of 840 (for a given energy of 50 keV), showing that the defect formation is promoted over the effects of the implanted impurity. Subsequent annealing at 1573 K was conducted in a home-made lamp furnace in an ambient atmosphere. The rate of heating was about 20 °C min^–1^ and the annealing duration was 10 min.

The macroscopic in-plane resistivity ρ(*T*) and mobility μ(*T*) were measured using the van der Pauw method coupled with the Hall effect (ECOPIA HMS-5000). Two measurement setups were used: a low-temperature cryostat, from 80 to 350 K, and a high-temperature measurement setup, from 300 to 750 K. All of these measurements were performed using a constant magnetic field up to 0.580 Tesla. The rate of temperature increase during measurements was close to 3 °C min^–1^.

Optical measurements were carried out using a J. A. Woollam M2000XI ellipsometer in the range of 0.2–1.7 μm. Ellipsometric data were acquired at 55, 65, and 75° angles of incidence. To determine the optical properties, a three-oscillator model was developed using J. A. Woollam CompleteEase software: a Tauc–Lorentz oscillator (TLO) centered close to 2.4 eV, modeling the direct band-gap absorption of ScN; a Gaussian oscillator (GO) arbitrarily centered out of the measurement range at 7 eV to model all UV interband transitions; and a Drude oscillator (DO), modeling the free-carrier optical behavior in the NIR range. The optical properties of the Al_2_O_3_ substrate were modeled using the optical constants available in the J. A. Woollam database.

X-ray diffraction (XRD) measurements were performed on a four-circle diffractometer (Seifert Space XRD TS-4) with a Cu X-ray source using a 0.5 mm collimator and a Meteor0D detector. The residual stress was analyzed using the sin^2^ Ψ-method, which relies on the use of lattice plane spacing *d*_*hkl*_ as an internal strain gauge. Along a given direction (Ψ, Φ), where Ψ is the angle between the surface normal and the normal to (*hkl*) planes and Φ is the azimuthal angle, the measured lattice strain ε_Ψ,Φ_ is given by
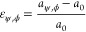
1where *a*_0_ is the stress-free lattice parameter and *a*_Ψ,Φ_ is the lattice parameter determined from a given {*hkl*} reflection. The *a*_0_ parameter is generally unknown and might differ significantly from the *a*_bulk_ parameter, preventing a direct determination of the strain.

Transmission electron microscopy (TEM) data were acquired using a TALOS F200S Thermofisher microscope operating at 200 kV. Slices, with a thickness of about 300 μm, were cut from the bulk sample. They were then prethinned up to a thickness of approximately 20 μm by mechanical polishing and glued onto a copper grid. Finally, ion thinning down to electron transparency was performed by means of a Precision Ion Polishing System (Gatan-PIPS). A TEM thin foil was also extracted by focused ion beam (FIB) Helios G3 CX from Thermofisher Dual Beam. The lamella was cut perpendicular to the basal plane, and was about 12 μm long, 4 μm wide, and 80 nm thick (approximate values).

The in-plane Seebeck coefficient and the electrical resistivity were measured simultaneously under a low-pressure helium atmosphere using an ULVAC-RIKO ZEM3 from RT up to 680 K.

Thermal conductivity measurements were performed at room temperature by a frequency domain thermoreflectance (FDTR) setup. This technique is a noncontact and nondestructive optical method. The measurement of the temperature oscillation induced by the absorption of an intense modulated laser beam (pump) allows its thermal characterization. A thin metallic film (Aluminum, 67 nm) was deposited on top of the sample to confine the heat absorption and to sense the surface temperature by its reflectance change. The modulated CW pump laser is focused on top of the surface of the sample. The absorbed energy induces a periodic temperature change. A second CW laser beam (probe) overlaps the heating area and is reflected to a photodiode. The relative variation of the probe intensity (Δ*I*/*I*_0_) is detected with a lock-in amplifier synchronized on the pump frequency. The pump frequency (*f*) is swept from a few kHz up to 100 MHz and the best fit of the spectral response is then performed with a multilayered model. The thermal conductivity (*k*_total_), the heat capacity (*C*_TH_), and the thermal contact resistance (*R*_contact_) are thus obtained.

## 3 Results

### 3.1 Structural Characterization

**Figure 2:**
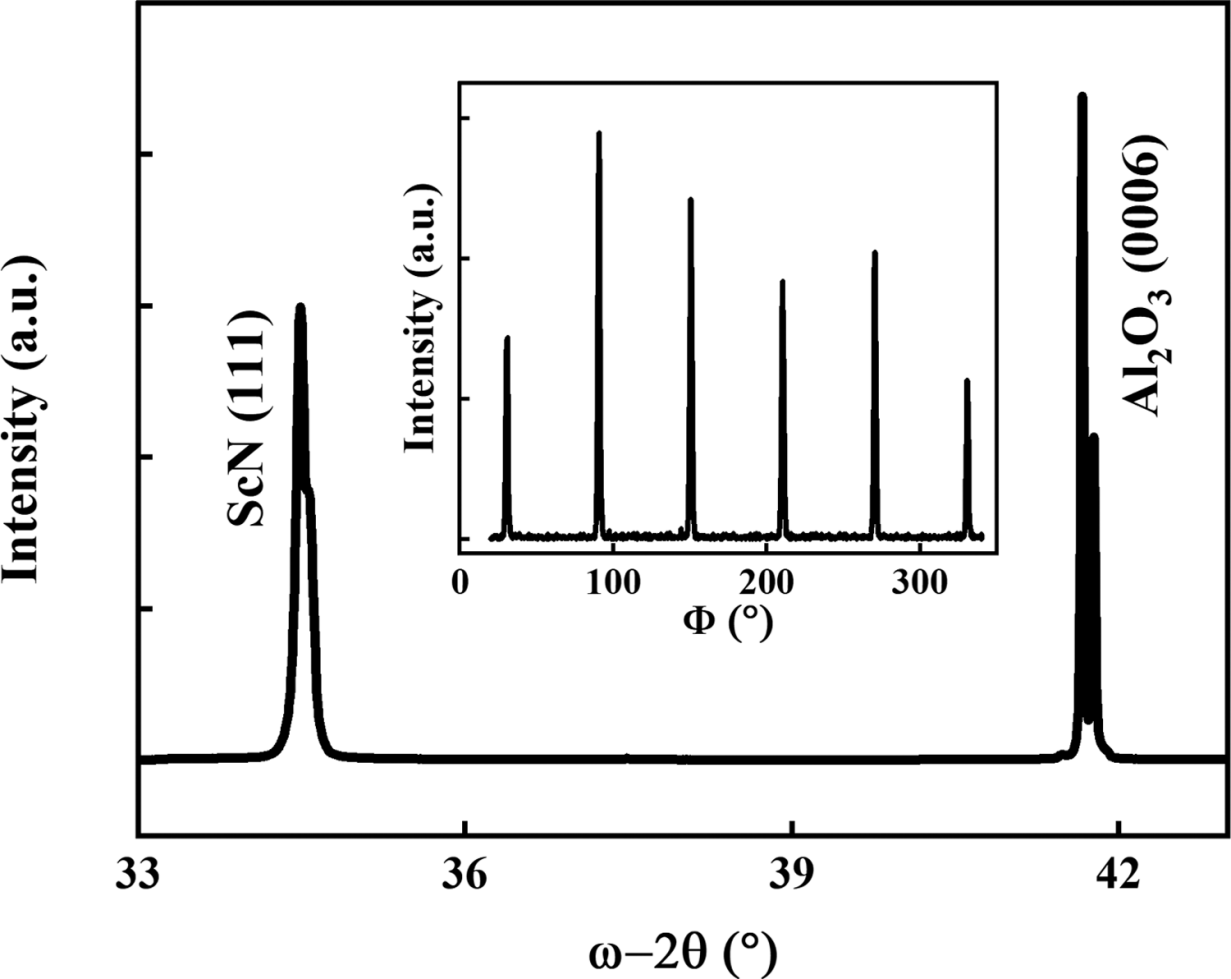
ω–2θ X-ray diffraction pattern (off set 0.2°) from a ScN(111) film deposited onto an Al_2_O_3_ (0006) substrate. The inset shows a Φ scan at Ψ =70.5°.

Figure 2 shows a ω–2θ (off set 0.2°) XRD scan of the as-deposited ScN thin film on a sapphire substrate. The peak observed at 34.5° is identified as (111) ScN with a lattice parameter of 4.50 Å, in good agreement with ICCD PDF 00-045-0978 (ScN). The inset shows the ϕ scan at an azimuthal angle Ψ = 70.5° for ScN {111}. The presence of six peaks shows that the ScN thin film grows epitaxially in the [111] out-of-plane direction with the presence of twin domains usually observed for the growth of a cubic material system on *c*-plane sapphire.^13,23^

**Figure 3:**
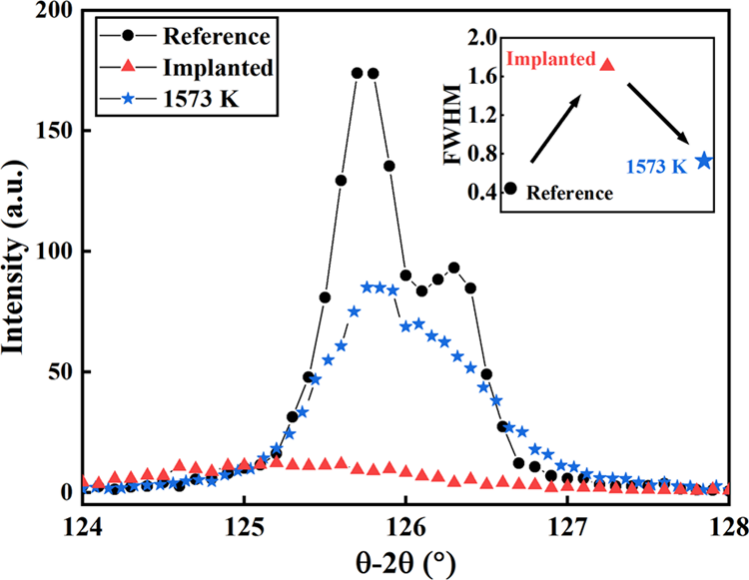
θ–2θ X-ray diffraction pattern of the ScN(333) peak after different steps of investigation of samples (reference, Ar-implanted, and annealed at 1573 K).

Figure 3 compares the diffraction peaks of the 333-diffraction peak for the as-deposited (reference), implanted, and annealed ScN samples. Even present at a high 2θ angle, the 333 reflection was chosen to observe more clearly any changes (shift, shapes, intensity) compared to other lll reflection. As seen, the reference sample exhibits the two peaks from the nonmonochromatic X-ray source (*K*_α1_ and *K*_α2_), showing the good crystallinity of the film. After implantation, a large drop in intensity is observed and the *K*_α1_ and *K*_α2_ split is no longer visible, suggesting a drastic change of the structure. The full width at half maximum (FWHM) is also strongly enlarged, indicating that the local strain heterogeneities are increased by the implantation. A shift toward the lower 2θ angles is observed, indicating that the interplanar distance *d*_333_ is expended by the implantation. The subsequent annealing at high temperature (1573 K) results in the partial restoration of the structure of the sample.

The effect of implantation-induced damage was studied by quantifying the residual stresses applied to the film. The planes, not parallel to the surface, 333̅ were studied (at Ψ of 70.53°) and compared to the 333 reflection; the data are summarized in Table 1. For the reference sample, the interplanar distances are almost equal between the growth direction and the in-plane direction showing that no (or few) residual stress is present. On the contrary, implanted and annealed samples have different values of *d*_333_ and *d*_333̅_. After implantation, *d*_333_ > *d*_333̅_ implies that the film undergoes compressive stress due to its expansion after the ion implantation. The same conclusion can be drawn from the stress values evaluated from the “sin^2^ Ψ-method”. The stress obtained after implantation is negative, showing the drastic change in the structure and the induced compressive stress. After annealing, the residual stress is found to be positive and the relation *d*_333_ < *d*_333̅_ is observed, indicating a strong recovery of the damage and thus a partial restoration of the film structure. However, the value of the residual stress is higher and leads to compressive stress compared to the one from the reference film, suggesting that annealing has indeed restored the structure and in addition to recombination, a defect evolution has occurred during high-temperature annealing.

**Table 1:** X-ray Diffraction Data for the ScN Thin Films As-Grown (reference), Ar-Implanted (5 dpa), and Annealed (1573 K)

samples	*d*_(333)_ (Å)	*d*_(333̅)_ (Å)	σ (GPa)	*a*_0_ (Å)
reference	0.8656	0.8654	0.090 ± 0.05	4.496
Ar-implanted	0.8681	0.8670	–0.320 ± 0.05	4.507
annealed 1573 K	0.8654	0.8661	0.270 ± 0.05	4.497

**Figure 4:**
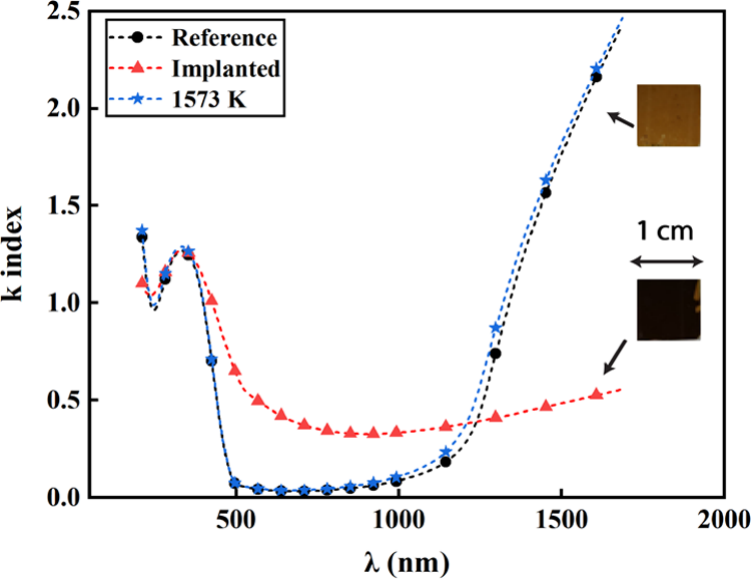
Extinction coefficient k of the ScN film at different steps of the process: reference, Ar-implanted, and annealed at 1573 K. The optical appearance of the film is presented on the right side.

The analysis of the optical properties (refractive index n and extinction coefficient *k*) of conductive materials gives access to local electrical properties, i.e., the in-grain mobility and carrier density.^24^Figure 4 shows the variation of the optical extinction coefficient *k* versus the incident wavelength. The curve of the reference ScN sample shows different domains: a transparent region from 500 to 900 nm in-between two absorbing regions the inter- and intraband absorptions. These wavelength ranges are in good agreement with those already measured and calculated using density functional calculations onto single-crystal ScN on MgO(001).^25,26^ Starting at 900 nm, the curve is representative of Drude’s model, suggesting a metalic-like behavior of ScN. Using an effective electron mass of 0.40*m*_0_, the mobility inside the grain at RT has been determined to be μ_grain_^ref^ (RT) ∼ 35 cm^2^ V^–1^ s^–1^ and the carrier concentration is about 2.1 × 10^21^ cm^–3^.^25^ After implantation, the *k*-curve is drastically altered in agreement with XRD (Figure 3). The transparent domain has disappeared, and the electrical nature of the film is nearly lost due to the as-introduced defects preventing any Drude analysis and thus any determination of the electrical properties. The subsequent annealing at 1573 K leads to the recovery of the optical constants and the sample tends to recover its initial color. The carrier concentration is found to be fully restored in contrast to the mobility, which is estimated to be μ_grain_^1573 K^ (RT) ∼ 28 cm^2^ V^–1^ s^–1^. The optical measurements confirmed that some defects are still present within the ScN grains even after annealing.

**Figure 5:**
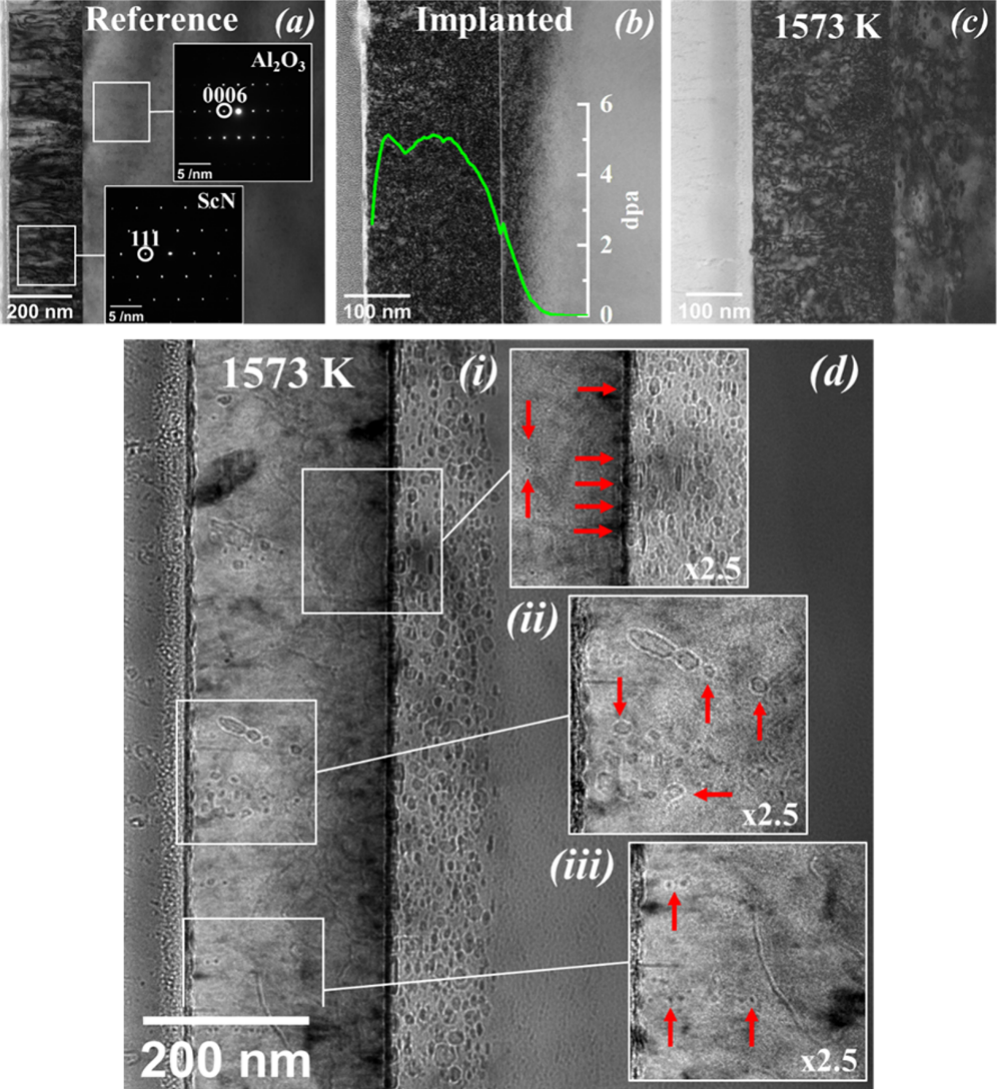
Bright-field (BF) cross-sectional TEM micrographs of the ScN film on the Al_2_O_3_ (0001) substrate for the reference (a), Ar-implanted (b), and annealed sample at 1573 K (c). (d) Detailed BF image of the annealed sample: tilted with α = −16° and β = 0.83° and overfocused. The insets highlight the interface region (i), large (ii), and tiny bubbles (iii). Bubbles are marked with arrows.

Figure 5 shows the TEM analysis of the ScN reference sample along with the implanted and the implanted/annealed one. Figure 5a shows an overview cross-sectional TEM image of a typical ScN film. Columnar domains are visible, highlighting the highly textured polycrystalline character of the film. A thickness of 240 nm can be estimated from the image, in good agreement with the X-ray reflectometry (XRR) measurements. After implantation, the implanted zone is clearly visible, as shown in Figure 5b. This zone comprises black contrast dots uniformly distributed up to a depth of about 300 nm, in agreement with the SRIM simulations (superimposed in the figure). These small dots cannot be resolved individually and suggest the formation of clusters of defects (interstitials). No other type of defects is observed. Energy-dispersive X-ray spectroscopy-scanning transmission electron microscope (EDS-STEM) mapping showed the presence of dispersed argon in the film and beyond into the substrate as expected from SRIMS simulations (Figure 1).

The TEM image, Figure 5c, attests to a change of the microstructure happening after annealing at a high temperature of 1573 K on an ion-implanted sample. First, there is the formation of large argon-filled cavities (bubbles evidenced by EDS data) as well as dislocations in the implanted area of the substrate (inset (i)). Most bubbles are faceted along basal planes; the others, larger ones, are rather spherical. These observations are similar to those obtained after helium implantation performed on an Al_2_O_3_ single crystal.^27^ In the ScN film, the microstructure looks different. There are fewer black contrast dots than before annealing, revealing that the columnar structure of the film is still preserved despite all of the processes. Bubbles in the ScN film are also observed, but they are heterogeneously distributed. Most appear spherical and small, a few nanometers in size (inset (iii)), while some defects with a more elongated shape are highly dispersed and can reach 30–40 nm (inset (ii)). It would also appear that many bubbles are present at the film/substrate interface, resulting in an almost bubble-free zone of about 50 nm inside the film, see the inset (i) in Figure 5d.

### 3.2 Physical Characterization

**Figure 6:**
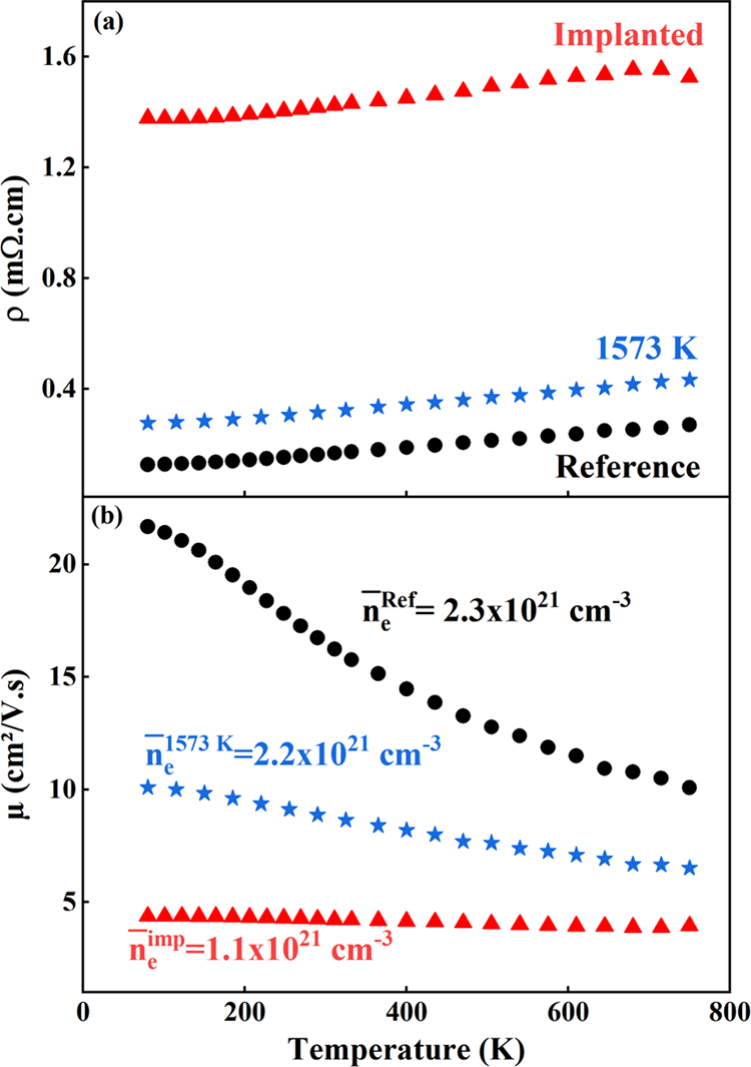
Resistivity (a) and mobility (b) as a function of the temperature of the reference, Ar-implanted, and annealed ScN thin films.

The electrical resistivity measurements are plotted in Figure 6a in the operating temperature range of 80–750 K. All curves show a similar appearance characteristic of metallic-like behavior. With increasing temperature, the electrical resistivity exhibits a constant value from 80 K up to 150 K approximately, and then a linear positive increase. The absence of any thermally activated transport at low temperature suggests an electrical resistivity controlled by impurities and the as-grown crystal defects. In the linear region (*T*_0_ > 150 K), the resistivity can be given by the following relation
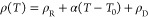
2where ρ_R_ is the residual resistivity and ρ_D_ is the implantation-induced resistivity (ρ_D_^ref^ = 0). As observed, the slope of curves α ∼ 2.2 × 10^–7^ ± 0.1 Ω·cm·K^–1^ is found to be constant at any stage of the process, indicating that the electron-phonon interaction is not modified by the implantation-induced defects and their evolution upon annealing at 1573 K: only ρ_D_ changes. For the 5 dpa Ar implantation, ρ_D_^implanted^ = 1.25 × 10^–3^ Ω·cm is found to be temperature independent in all of the investigated temperature ranges, and no recovery of damage or defects recombination occurs during the electrical measurements. This suggests that all of the defects produced by the Ar implantation are stable up to at least 750 K, in contrast to what was observed for Mg-dopant implantation in ScN.^18^ An annealing at 1573 K restored the electrical resistivity to comparable values as the reference film, and the value of ρ_D_^impl^ is reduced by about 90%.

Figure 6b shows the Hall mobility curves with the temperature that helps to understand the carrier scattering mechanism and reports the average of carrier concentrations. According to Matthiessen’s rule, the total mobility can be written as
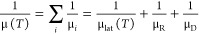
3where μ_lat_ is the lattice mobility, μ_R_ is the residual mobility, and μ_D_ the implantation-induced defect mobility. For the reference ScN, μ_D_^ref^ is taken as ∞. The Hall mobility curve for the reference sample decreases smoothly with temperature, but more slowly than expected for acoustical lattice scattering for which the relationship is μ_lat_ (*T*) ∼ *T*^–1.5^ (or *T*^–1.29^, as reported in MBE-ScN^28^). It can be well fitted by adding the carrier scattering by residual impurities (as-grown defects and grain boundaries) taken as μ_R_ ∼ 23 cm^2^ V^–1^ s^–1^ for *T* > 150 K. The Hall electron mobility strongly reduced by the argon implantation seems to be constant with temperature (80–750 K), μ^implanted^ (*T*) = 4–5 cm^2^ V^–1^ s^–1^, with a major contribution of defects’ mobility estimated at μ_D_^implanted^ ∼ 5 cm^2^ V^–1^ s^–1^ using eq 2. Annealing did not fully restore the total mobility showing the partial recovery of defects and leading to an increase of μ_D_^1573 K^ ∼ 18 cm^2^ V^–1^ s^–1^ (*T* ∼ 300 K).

The carrier concentrations of samples are found to be high and independent of temperature, which is a typical trait of degenerate semiconductors. The Ar implantation in this highly damaged regime reduced by a factor of two of the free-carrier concentration, showing that the as-introduced defects act like traps for electrons. In contrast, the carrier concentration was almost recovered during annealing (carrier detrapping). However, defects are still present after annealing and affect the charge carrier mobility while being no longer electrically active. These electrical measurements are in good agreement with the optical characterizations, namely, a fully recovery of the carrier concentration after annealing and a partial recovery of the carrier mobility.

**Figure 7:**
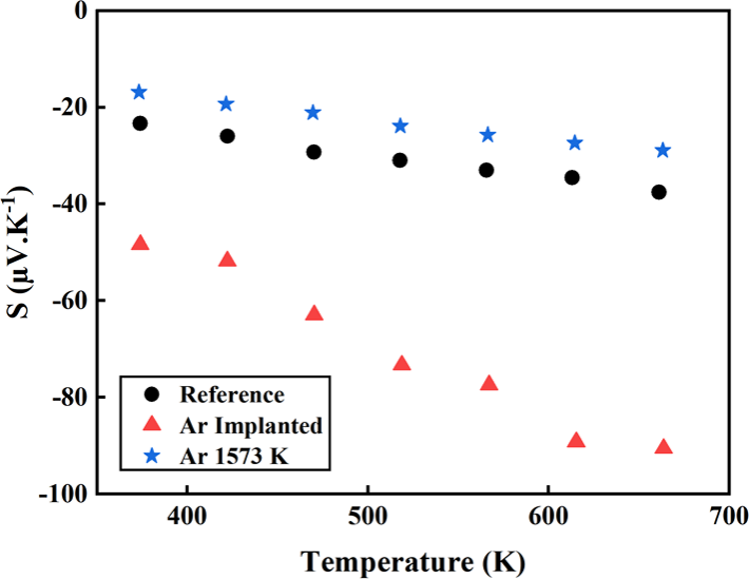
Temperature dependence of the Seebeck coefficient (S) from 370 to 680 K of the reference, the Ar-implanted (5 dpa), and annealed (1573 K) samples.

Seebeck coefficients measured in the temperature range of 370–680 K are displayed in Figure 7. |*S*| increases linearly with the measuring temperature for all of the samples, regardless of the process step. The linear dependence with temperature suggests a constant carrier concentration in agreement with Hall effect measurements (Figure 6b) At 600 K, the value of |*S*| for the reference ScN is 35 μV·K^–1^, being close to the value previously reported for samples produced under similar conditions but lower than the one reported for ScN growth on a MgO substrate using MBE.^15,18^ This low value may be due to the large contamination from impurities acting as dopants, resulting in the large carrier concentration measured. After implantation, the Seebeck coefficient absolute values in the high damage regime increases from 30 to 85 μV·K^–1^ at 600 K. As observed, the implantation process also increases the slope of the curve by a factor close to three. After annealing at 1573 K, the absolute value of the Seebeck coefficient is found to be slightly lower than the reference value, ∼ 28 μV·K^–1^ at 600 K, with, however, the slope back to the same value. The Seebeck coefficient must therefore be analyzed considering both the number of carriers and the presence of defects that change the density of states (DOS).

Table 2 reports the values of the thermal conductivity of ScN measured at 300 K, the heat capacity of the ScN thin film, and the interface thermal resistance between the top aluminum film and the layer of interest. The thermal conductivity value of the reference sample is 12.5 W·m^–1^·K^–1^, in good agreement with the values reported earlier.^14,18^ After Ar implantation, the sample exhibits a large drop of thermal conductivity down to 3 W·m^–1^·K^–1^. As previously mentioned, the annealing process afterward restored partially the structure and the defects, leading to a thermal conductivity toward its original value (around 8.5 W·m^–1^·K^–1^) and highlighting again the presence of structural defects even after high-temperature annealing. The heat capacity is not affected by the entire process. The contact thermal resistance increases slightly after implantation and it recovers a value comparable to the reference sample after annealing.

**Table 2:** Identified Thermal Conductivity, Contact Resistance, and Heat Capacity for Reference, Ar-Implanted, and Annealed ScN Thin Films

samples	reference	Ar-implanted	annealed 1573 K
*k*_total_ (W·m^–1^·K^–1^)@300 K	12.5	3	8.5
*R*_contact_ (nK·m^2^·W^–1^)@300 K	18	24	14
*C*_TH_ (MJ·K^–1^·m^–3^)@300 K	4.07	4.03	4.1

## 4 Discussion

The large decrease of thermal conductivity in implanted ScN thin films either with noble gas (Ar in this study, see Table 2), with dopants (Mg in a previous study), or by nonelectrically active element (Li^+^) may be explained by the as-introduced defects, which reduce the mean phonons free path, increasing thus the level of scattering.^18,19^ The increase of thermal conductivity occurring during the subsequent high-temperature annealing suggests a partial recovery of defects. However, the Seebeck curves and electrical characterizations provide a more complete picture. The implantation defects introduce a deep acceptor level in the band gap and then reduce the concentration of free carriers. Moreover, this should modify locally the electronic DOS. For degenerate semiconductors, the Seebeck coefficient is dependent on the effective mass of carriers at the Fermi surface.^7^ Besides, calculations showed that vacancies introduce an asymmetrical peak close to the Fermi level in the electronic DOS of ScN, resulting in an enhancement of the Seebeck coefficient.^29,30^ As a result, the trapping of carriers and the modification of the DOS caused by the implantation process led to an increase of both the Seebeck value and the slope (x3 in the implanted sample) of the curve *S*(*T*) (Figure 7), in the whole investigated temperature range. This increase in the slope is also observed in the previous study when implanting Mg dopants in ScN.^18^ A higher *S*(*T*) slope was reported when measuring the thermoelectric properties up to the temperature at which the defect recombination starts to be active (any zero-dimensional defects such as the Frenkel pairs), i.e., at 450 K.^18^ This triggering of defect recombination results also in a progressive decrease of electrical resistivity during the measurement. This behavior was reported regardless of the concentration of implanted Mg.

In the present paper, no change in the slope is observed when implanting argon at high fluence (Figure 6); the Ar atoms, therefore, operate as point defect stabilizers, preventing any defect recombination (or damage recovery) at least up to a temperature of 750 K (see the electrical resistivity and Seebeck curves in Figures 6 and 7; no modifications occur in the temperature range). Noble gas (NG) atoms are known to behave singularly when implanted in materials.^20^ Because of their low solubility in materials, they tend to aggregate, resulting in the formation of NG-extended defects such as cavities or highly pressurized bubbles in fluid or in solid form depending on implantation conditions.^31^ Electronic structure calculations in SiC and Si showed that the trapping of argon (as other heavy gases) by mono- or divacancy is energetically favorable.^32,33^ Implanted argon atoms in ScN are thus expected to be trapped by the supersaturation of vacancies introduced by the collision cascades. Thus, during the implantation process, many interstitials recombine with vacancies (dynamical annealing) or combine with others to form interstitial clusters that appear as black spot damage in TEM (Figure 5a). All of the remaining vacancies trap the argon atoms to form argon vacancy complexes, Ar_*n*_*V*_*m*_ with *m* > *n*. Up to 750 K, neither the interstitial clusters nor the Ar_*n*_*V*_*m*_ complexes have sufficient energy to dissociate, and no change in the slopes of the Seebeck and resistivity curves is reported with the measuring temperature (Figures 6 and 7).

All of these defects contribute predominantly to the scattering of the free carriers and therefore reduce drastically the electrical mobility. After annealing, the recovery of the Seebeck slope shows the removal of the DOS changes caused by the implantation. The values of |*S*| are however slightly lower than in the reference sample, highlighting the change in the type of defects. Moreover, this evolution is coupled with a change in the stress state of the film from compressive to tensile.

The different as-introduced defects have an influence on the mobility of free carriers by reducing their free mean path. The black dot damage observed after implantation suggests that the primary knock-on-atoms go on to generate collision cascades in which Frenkel pairs are formed. Probably, due to the low migration energies, if many interstitials recombine with vacancies, others combine to form clusters appearing as black spot damage on the TEM image, as shown in Figure 5b. According to the temperature of subsequent annealing, such clusters have sufficient energy to dissociate, giving rise to a lower density of larger black spots, as shown in Figure 5c when compared to Figure 5b. The continuous recovery of the resistivity with annealing suggests a size dependence of the dissociation energies of the clusters. The role of Ar- gas leads to the stabilization of vacancy-type defects as cavities (bubbles or voids) is observed before annealing. Upon annealing, the TEM observations suggest that some of the (Ar_*n*_*V*_*m*_) complexes dissociate/migrate to other complexes until they form visible bubbles. However, their formation and growth appear to be the result of a combination of several factors including stress evolution, film structure (as the presence of grains), and defect mobility, resulting in a rather heterogeneous distribution of bubbles in the film. As an example, the growth of bubbles in SiC is found to be enhanced on grain boundaries.^34^ In ScN, by applying Matthiessen’s rules to optical and Hall mobilities at RT, the mobility due to grain boundaries is found to be reduced after implantation and annealing from μ_GB_^ref^ (RT) ∼ 35 cm^2^ V^–1^ s^–1^ to μ_GB_^1573 K^ ∼ 13 cm^2^ V^–1^ s^–1^. This shows that the grain boundary scattering is not neglectable for carrier mobility and that an accumulation of defects also occurs on the grain boundaries during the 1573 K annealing. Similarly, the interface (14% lattice mismatch) appears to act as a sink for bubble formation. TEM observations are still in progress to obtain a clearer picture. All of these defects will therefore influence the transport properties of ScN.

Combining the Seebeck and electrical conductivity values, the power factor at 600 K for the reference sample is found to be about 5 × 10^–4^ W m^–1^ K^2^. This value is relatively low compared to the previous studies on as-grown and Mg-implanted ScN due to the quality of the film,^13,14,18^ i.e., the unwanted dopant impurities introduced during the film synthesis as the amount of oxygen or fluorine. The quality of the ScN film is a critical factor in improving its thermoelectric properties. While implantation damage leads to a strong increase in the value of the Seebeck coefficient, it also strongly reduces the electrical conductivity, which, in turn, has no or few effects on the calculated value of the power factor, ∼5 × 10^–4^ W m^–1^ K^2^. On the contrary, the damage induces a strong reduction in thermal conductivity, which is beneficial for improving the thermoelectric figure of merit of ScN, and these defects are stable to at least 750 K. Postimplantation annealing results in an evolution of defects, leading to a detrapping of free carriers, but these defects (interstitial clusters, bubbles, vacancies-gas complexes: 3D defects) still affect the mobility and also reduce the thermal conductivity, which was the purpose of this study. Then, knowing that the types of defects are strongly dependent on the implantation and postprocessing conditions, these must be optimized to find the best balance between all of the interrelated parameters (*S*, ρ, and *k*_total_).

## 5 Conclusions

Argon implantation at room temperature in a high regime of damage (5–6 dpa) was carried out on a ScN thin film to introduce defects to reduce the lattice thermal conductivity. All implantation defects created are stable up to a minimum operating temperature of 750 K: the use of argon, therefore, stabilizes implantation-induced defects. These defects act as carrier traps, reduce their mobility, and also have a strong effect on the thermoelectric properties of ScN. In particular, the thermal conductivity is found to be reduced by a factor of four while keeping the PF constant. Postimplantation annealing at high temperature restores the crystallinity of the ScN structure and the number of free carriers but also leads to the formation of nanosized 3D defects, which both affect carrier mobility and phonon scattering. However, these defects have a detrimental effect on the power factor, showing that the thermoelectric properties are strongly dependent on the size, morphology, and type of defects.

Thus, this study shows that the controlled introduction of defects, or defect engineering (via noble gas implantation for thin films), can be used as a strategy to introduce additional phonon scattering centers that are beneficial for the development of TE materials.
