# Correction to Influence of Generated Defects by Ar Implantation on the Thermoelectric Properties of ScN

**DOI:** 10.1021/acsaem.3c00252

**Published:** 2023-02-15

**Authors:** Razvan Burcea, Jean-François Barbot, Pierre-Olivier Renault, Dominique Eyidi, Thierry Girardeau, Marc Marteau, Fabien Giovannelli, Ahmad Zenji, Jean-Michel Rampnoux, Stefan Dilhaire, Per Eklund, Arnaud le Febvrier

In the description of Figure 6, in the text after eq 2, the sentence “*This suggests that all of the defects produced by the Ar implantation are stable up to at least 750 K, in contrast to what was observed for Mg-dopant implantation in ScN.*^18^” should be corrected to “*This suggests that some defects produced by the Ar implantation are stable up to at least 750 K, and this could have been expected if the resistivity measurements had been repeated on the results observed for Mg-dopant implantation in ScN.*^18^”

The reason for this correction is because of contact problems during the resistivity measurement; all implanted samples had reached 770 K (497 °C) prior to performing the final measurement presented in the main work. That heat treatment may have resulted in recombination of some defects, as observed by Tureson et al. (see ref 18) during the thermoelectric properties measurement.

This does not alter the remaining results, discussion, and conclusions presented in this work.

